# Analysis of Spatial Pattern Evolution and Influencing Factors of Regional Land Use Efficiency in China Based on ESDA-GWR

**DOI:** 10.1038/s41598-018-36368-2

**Published:** 2019-01-24

**Authors:** Xiaoshu Cao, Yongwei Liu, Tao Li, Wang Liao

**Affiliations:** 1 School of Geography Science and Planning, Sun Yat-sen University, Guangzhou, 510275 China; 2 Institute of Transport Geography and Spatial Planning, Shaanxi Normal University, Xi’an, 710119 China

## Abstract

In order to give an in-depth understanding of the contradictions arising from the land resource supply and demand, this study selected 30 provinces (some are autonomous regions or municipalities) in China to be the research unit, used the carbon emission as an undesirable output, and adopted the Super-SBM DEA model and ESDA-GWR method to research the evolution characteristics and influencing factors of land use efficiency in China in 2003–2013. The results indicated that: (1) The land use efficiency in China overall was moderately ineffective and the overall utilization level was low; (2) The Global Spatial Autocorrelation was instable and had maintained a high level; (3) The “hot spots” mainly being distributed in the southeast coastal regions and “cold spots” being found in the central and western regions, so that as time goes on, the pattern of “high in the east and low in the west” has been gradually formed and stabilized. (4) The GWR model analysis showed that the natural factors such as NDVI, DMSP/OLS and DEM have a significant impact on land use efficiency, thereby providing an important contribution to this study. For the eastern coastal areas, the emphasis should be improving their OT, PF and PGDP, for the western region, should focus on improving its comprehensive economic development level to improve the DMSP/OLS, while strengthening the ecological environment to improve the level of NDVI.

## Introduction

How to economically and intensively utilize the finite land resource has been the core issue in China’s economic and social development. In 2014, China approved the construction land of 403,800 hectares and approved the farmland occupation about 160,800 hectares, resulting in a sharp decline in China’s arable land stock. As the social economy of China develops, the contradiction arising from the land supply and demand has gradually been intensified, posing challenges for the sustainable economic and social development. Many scholars have studied the agrarian problems in China^1–4^. Therefore, implementing a very stringent strategy for economical and intensive land use –in other words, to increase the land use efficiency – is important for resolving the contradiction arising from the land resource supply and demand. Clearly, it is of great practical significance to explore the spatio-temporal variation characteristics of land use efficiency and its factors.

Land use efficiency refers to the increase in the output of a unit land area with respect to regional social and economic activities. It is not only related to the efficient use of land resources, but also is the essential foundation for the sustainable development of urban regional systems. For some time, the land use efficiency has been an important topic for scholars in China and elsewhere. The early researches on land use efficiency mainly focused on the urban land use, the theoretical modeling of urbanization, and the urban management^5–8^. The current researches primarily focused on the functions and operational mechanisms of the land market, the land property rights and the allocation efficiency, the land use efficiency evaluation and application, etc.^9–16^. According to the research results of efficiency evaluation of city land utilization in the country, urban agglomerations and different cities, scholars have conducted extensive and deep researches on urban land use efficiency in China. This research mainly studies the basic theory of urban land use efficiency, the evaluation index systems, the model construction and evaluation methods, the comprehensive utilization effectiveness, and the ways for improving the urban land use efficiency^17–21^.

The research on land use efficiency tends to be gradually perfected in respects of methods, models and indexing systems. Among them, DEA has been widely used in empirical research, but most of the studies were based on the traditional DEA. In addition, the perspectives of the spatial correlation and the heterogeneity were seldom involved. The existing research methods gradually use the qualitative analysis instead of the quantitative analysis, such as the regression statistical analysis, the data envelopment analysis and the spatial analysis method, and the existing research method has fully integrated the mainstream technology methods and spatial analysis trends. However, most researchers mainly use the traditional DEA method to evaluate the land use efficiency, which generally is based on the economic and social development of the region. A research which only studies the influential factor namely the natural environment on the land use efficiency in a particular area is far not enough. Based on this, the outstanding feature of this study is using the Super-SBM DEA method, which has more advantages than the traditional method and can evaluate the land use efficiency more deeply. Furthermore, it can take into account the importance of both the social and natural factors.

Here, in this paper, it intends to understand the evolution of land use efficiency and influence factors in China. The goal of this study is to analyze the evolution of land use efficiency by using the Super-SBM DEA model and the ESDA-GWR method and is to list the influential factors for land use efficiency based on GWR. Upon the study of the undesirable output of carbon dioxide, the influence of natural and socioeconomic factors also is analyzed. The development of economy and society in China has been permeated with the contradictions when comes to the protection of resources and the environment, especially the land resource issue remains as a cardinal issue. The CO_2_ emission, as one of the outputs of pollutions, produces a negative impact, but making the land use efficiency model can more truly reflect the actual situation of China.

## Results

### Evolution of Land Use Efficiency Based on Super-SBM DEA and ESDA. In overall, it stays at a low level, but the regional differences are obvious

The results of the Super-SBM DEA model calculations for efficiency are shown in Table 1. Among them, the average score in 2013 was 0.623, which was only 50.76% of the optimal level (Shanghai had the highest score of 1.228). Nine provinces (30%) reached optimal levels of efficiency, while the remaining 21 provinces (70%) were sub-optimal, meaning that the overall land use efficiency was at a low level. In terms of the regional differences, the non-equilibrium of spatial differences was consistent with the level of economic development, which showed the spatial pattern characteristic of being high in the east and low in the middle and west of the country.

**Table 1:** Statistics on Land Use Efficiency in China.

Year	2003	2007	2010	2013
Minimum Value	0.234	0.243	0.241	0.227
Maximum Value	1.157	1.261	1.195	1.228
Average Value	0.613	0.616	0.635	0.623
Optimal (≥1)	Yunnan, Shanghai, Fujian, Guangdong, Beijing, Zhejiang, Tianjin, Liaoning, Anhui	Shanghai, Beijing, Fujian, Guangdong, Yunnan, Anhui, Tianjin, Zhejiang, Liaoning	Shanghai, Tianjin, Fujian, Beijing, Guangdong, Anhui, Yunnan, Zhejiang, Liaoning	Shanghai, Beijing, Tianjin, Yunnan, Anhui, Fujian, Guangdong, Zhejiang, Liaoning
Highly Ineffective [0, 0.25)	Guizhou, Gansu, Ningxia	Ningxia, Gansu	Ningxia, Gansu	Ningxia, Gansu
Moderately Ineffective [0.25, 0.5)	Qinghai, Shanxi, Inner Mongolia, Xinjiang, Sichuan, Shaanxi, Jilin, Henan, Hebei, Guangxi, Hubei, Heilongjiang, Jiangxi, Chongqing	Guizhou, Qinghai, Shanxi, Xinjiang, Shaanxi, Inner Mongolia, Sichuan, Chongqing, Henan, Guangxi, Hebei, Jilin, Hunan, Hubei, Jiangxi, Heilongjiang	Guizhou, Shanxi, Qinghai, Xinjiang, Sichuan, Henan, Guangxi, Inner Mongolia, Shaanxi, Hebei, Hubei, Jilin, Hunan, Chongqing	Guizhou, Xinjiang, Qinghai, Shanxi, Guangxi, Henan, Inner Mongolia, Shaanxi, Sichuan, Hebei, Hubei, Jilin, Hunan, Hainan, Jiangxi
Slightly Ineffective [0.5, 0.75)	Hunan, Shandong, Hainan	Shandong, Hainan	Heilongjiang, Jiangxi, Shandong, Hainan	Heilongjiang, Chongqing, Shandong
Close to Effective [0.75, 1)	Jiangsu	Jiangsu	Jiangsu	Jiangsu

Through the analysis, we found that the highest, lowest, and average values for each target year had an overall increasing trend. This paper divided the efficiency levels into five categories, highly ineffective, moderately ineffective, slightly ineffective, close to effective, and optimal. The results showed that the most common category was moderately ineffective, followed by optimal, highly ineffective, slightly ineffective, and close to effective-with a smaller distribution. The range of distributions in each area was large with clear polarization in efficiency. At the same time, regions of each type gradually tended to be stable, forming a clear distribution pattern of land use efficiency in China.

### Global Spatial Autocorrelation was Not Stable and Maintained a High Level

This study used GeoDa 1.6.7 software to calculate the global spatial autocorrelation index, with results shown in Table 2. The results showed that there was significant positive spatial autocorrelation for each year. In 2013, for example, the seven provinces that had optimal efficiency were mainly distributed throughout the eastern region, making up 77.78% of that region. In the central region, five provinces were moderately ineffective, which accounted for 83.33% of that region. In the west, nine provinces were highly or moderately ineffective. On the whole, the highest efficiency was in the east and the lowest was in the west. At the same time, the internal similarity within all three regions was very strong while the gap between them was extremely clear. Through the overall analysis of the Moran’s I values, we found that the global spatial autocorrelation was the lowest in 2007 and the highest in 2010, and it remained at a high level by 2013.

**Table 2:** Moran’s *I* Values for Target Years.

Year	2003	2007	2010	2013
Moran’s *I* Value	0.302	0.214	0.353	0.326
P value	0.006	0.004	0.005	0.003

### Area of Concentrated Types Mainly Consisted of “Hot Spots” and “Cold Spots” that are Generally Stable

**Figure 1:**
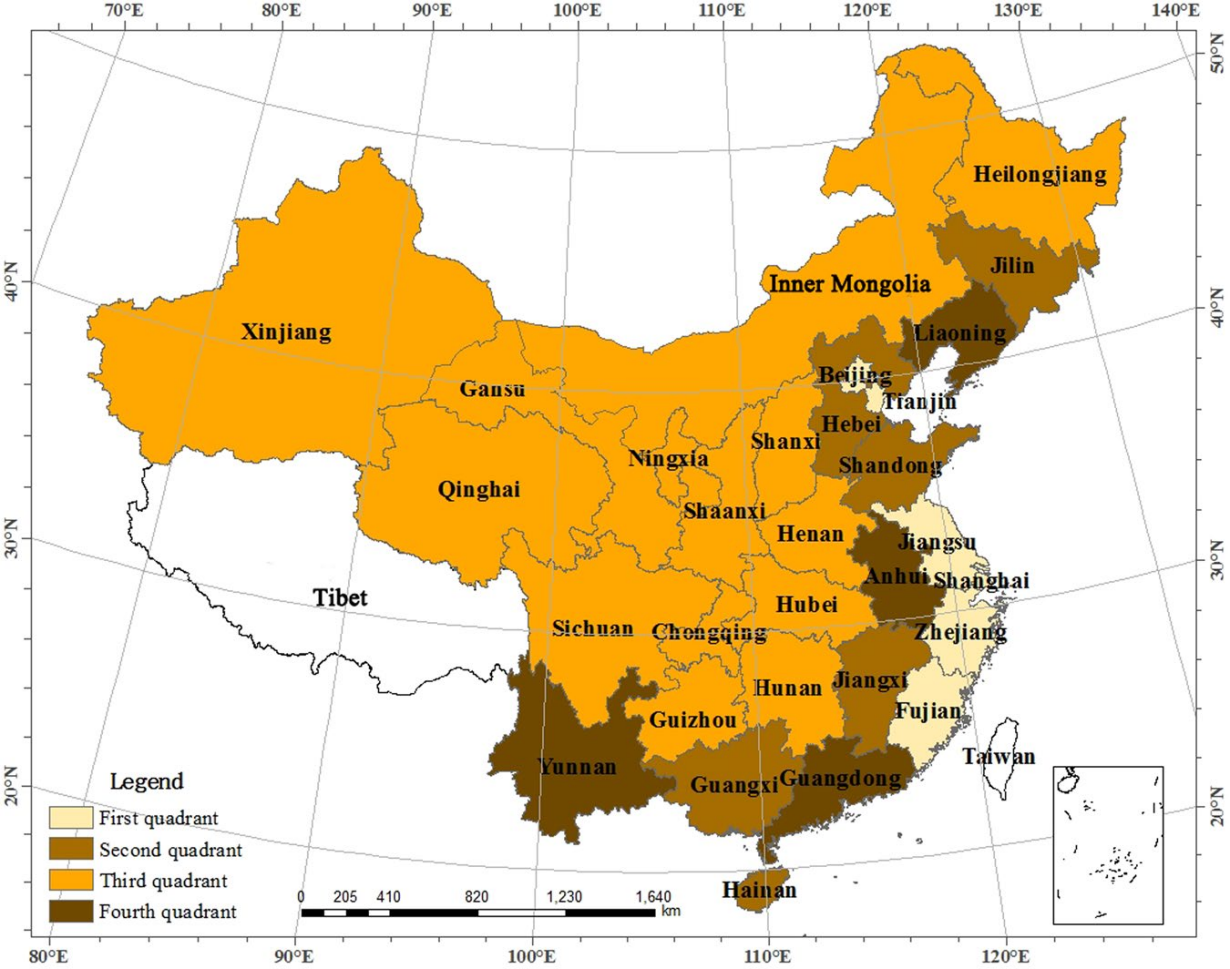
Distribution Map of the Provinces in The Scatter Plot in 2013. Map created using ArcMap (version10.2) software from Esri (http://www.arcgis.com/).

**Figure 2:**
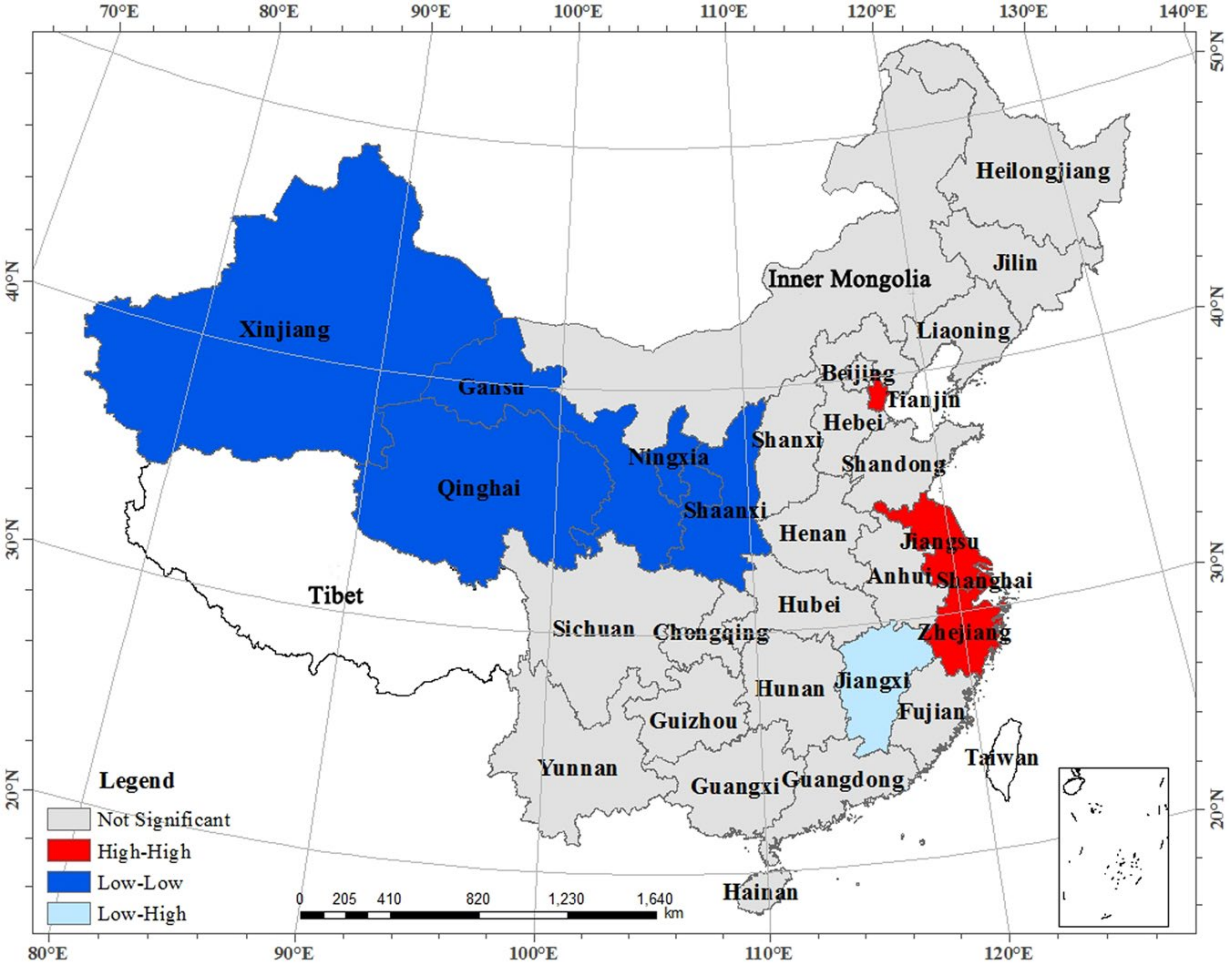
LISA Clustering Map in 2013. Map created using ArcMap(version 10.2) software from Esri (http://www.arcgis.com/).

GeoDa software was also used to calculate the local spatial autocorrelation index. Here was an example: the corresponding provinces within each quadrant for 2013 were labeled in a scatter plot, as shown in Figs 1 and 2. In Fig. 1, taking the standardized value of land use efficiency in each region as the abscissa and the weighted average (also called Spatial lag) of all neighboring provinces as the vertical axis draw scatter diagram, the provinces corresponding to each quadrant of the scatter plot were plotted. The first and third quadrants were spatially positively correlated and the second and fourth quadrants were spatially negatively correlated.

Through an assessment of Fig. 1, we found that most provinces were located in the first and third quadrant. There was a larger number of low value area clusters that were more widely distributed, which was the main driver of the positive spatial autocorrelation. Most of the “low-low” provinces in the third quadrant were located in the midwest, while all the “high-high” provinces in the first quadrant were located in the east-including Beijing, Tianjin, Jiangsu, Shanghai, Zhejiang, and Fujian. The “S” shape area, which was made up of Liaoning, Hebei, Shandong, Anhui, Jiangxi, Guangxi, Yunnan and Guangdong, formed the main distribution region of the second and fourth quadrant. Figure 2 shows the distribution of different provinces in 2013, and it shows that the overall performance of land use efficiency is mainly made up of the aggregations of “low-low” provinces, with few “high-high” provinces and fewer “low-high” and “high-low” aggregations. The aggregation trend of “hot spots” with high values is very clear, so is the “cold spots” with low values.

The statistics of the aggregation type of provinces in each target year distributed in the LISA map is shown in Table 3. Table 3 showed that provinces were included in each quadrant were relatively stable: the number of “low-low” aggregation was the largest, followed by the “high-high “aggregation, and both the second and the fourth quadrant had very few aggregations. The “high-high” were all located in the eastern provinces, the “low-low” were in the central and western provinces, only one province, Jiangxi, was found in the “low-high”, and only Yunnan Province was found in the “high-low” in 2010. Overall, the LISA aggregation area was relatively stable, and the “high-high” aggregation region presented an increasing trend. Among them, Jiangsu, Shanghai, and Zhejiang were adjacent to one another, which formed a “Hot spot” for high efficiency. The “low-low” aggregation region gradually was stabilized and was consisted by the vast areas of Xinjiang, Gansu, Ningxia, Qinghai, Shaanxi, and other western regions, which formed a “Cold spot” for low efficiency.

**Table 3:** LISA Cluster Types of Regional Land Use Efficiency in China.

Year	First quadrant (HH)	Second quadrant (LH)	Third quadrant (LL)	Fourth quadrant (HL)
2003	Jiangsu, Zhejiang, Fujian	Jiangxi	Inner Mongolia, Xinjiang, Gansu, Ningxia, Qinghai, Shaanxi	—
2007	Jiangsu, Zhejiang, Fujian, Shanghai	Jiangxi	Xinjiang, Gansu, Ningxia, Qinghai, Shaanxi, Chongqing	—
2010	Jiangsu, Zhejiang	Jiangxi	Xinjiang, Gansu, Ningxia, Qinghai, Shaanxi	Yunnan
2013	Jiangsu, Zhejiang, Tianjin, Shanghai	Jiangxi	Xinjiang, Gansu, Ningxia, Qinghai, Shaanxi	—

### Model building of GWR

GWR 4.09 software was used to carry out the geographically weighted regression and the Gauss function was used to construct the weighted function. The Cross-validation (CV) and the fixed kernel function were used to determine the optimal bandwidth. After calculation, the regression coefficient was 0.7159, and the overall fit was good.

### Influence factors analysis based on GWR

The regression coefficients for each explanatory variable are shown in Table 4. The absolute average value reflects the average contribution of each variable to land use efficiency. The sequence as follows: PGDP > NDVI > OT > PF > DMSP/OLS > PS > DEM, and only PS was negative, which demonstrated that an average increase in DEM, NDVI, OT, PF, DMSP/OLS, and PGDP led to an increase in land use efficiency, while the increase in PS led to a decrease. The further analysis of the maximum, Q1, median, Q3 and the minimum found that the coefficients for PGDP, PF, OT, DMSP/OLS, and NDVI were positive, which meant these variables also had positive effects on land use efficiency in each province. There were positive and negative values of DEM, which indicated it had positive and negative effects. PS were negative for all provinces, which produced a negative effect on land use efficiency in every province.

**Table 4:** Estimation Results for Land Use Efficiency Based on GWR. The full description for indicators such as PGDP, PS, PF, OT, DMSP/OLS, DEM and NDVI is in the chapter “Materials and Methods”, Min means the minimum value, Q1 means the 25% value of sorting all sample data from small to big, Median means the 50% value of sorting all sample data from small to big, Q3 means the 75% value of sorting all sample data from small to big, Max means the maximum value.

Variable	Average	Min	Q1	Median	Q3	Max
Intercept	0.617	0.605	0.612	0.616	0.621	0.630
PGDP	0.187	0.165	0.180	0.188	0.196	0.206
PS	−0.037	−0.066	−0.044	−0.035	−0.028	−0.021
PF	0.123	0.082	0.102	0.123	0.138	0.178
OT	0.127	0.112	0.123	0.128	0.132	0.140
DMSP/OLS	0.050	0.030	0.042	0.051	0.058	0.072
DEM	0.008	−0.052	−0.010	0.012	0.029	0.050
NDVI	0.139	0.128	0.133	0.139	0.143	0.154

**Figure 3:**
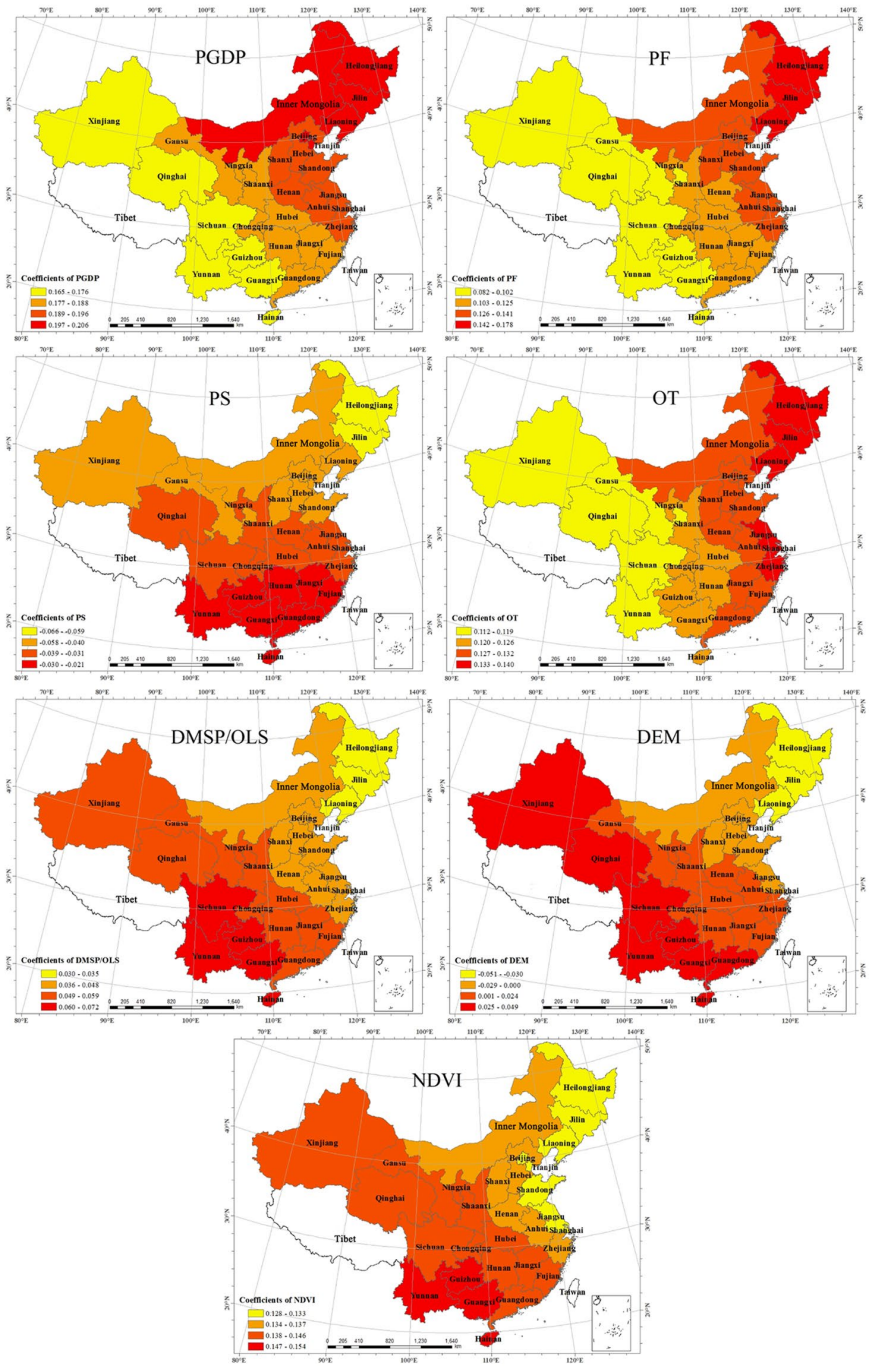
Distribution of the Regression Coefficient Based on GWR. Map created using ArcMap(version 10.2) software from Esri (http://www.arcgis.com/).

In order to analyze the influence of each variable on land use efficiency in each province, the coefficient distribution map for the influencing factors was created (Fig. 3). PGDP was an important indicator of the level of economic development, and its regression coefficient exhibited a characteristic of being “high in the northeast, low in the southwest.” The average PGDP value was the largest of the explanatory variables, indicating that improving the level of economic development was the most significant driver for improvement in land use efficiency. Based on the coefficient, the influence varied across the different provinces; among them, Heilongjiang was the most sensitive to this. When the PGDP changed by 1% the land use efficiency responded with a 0.25% change. The minimum influence was found in Yunnan Province, where a 1% change in PGDP led to a land use efficiency change only by 0.16%. On the one hand, with the continuous development of the regional economy, the mode of economic growth has been continuously transformed. The endogenous economic development and the high resource efficiency have improved the overall land use efficiency. On the other hand, however, with the continuous economic improvement, the contradiction arising from the supply and demand of land resource has been intensified. The economic and intensive use of the land resource, along with the improvement of the land stock utility potential, has become a key way to solve the contradiction arising from land supply and demand in the new era. With the continuous advancement and implementation of this strategy, the level of land use efficiency will be improved. The contradiction arising from the land supply and demand in eastern and northeastern part is greater than in the central and western part, so there is greater potential to improve the efficiency of land use by the economic and intensive land use.

PF was the main indicator of the regional investment level, which had a positive coefficient and basically the same distribution trend as PGDP. The main reason for this was that PF had a direct relationship with the land resource utilization pattern driven by the economic development and the stage of the economic development.

PS was an important indicator of the level of regional industrial structure, and its coefficient was negative. PS was the only variable that had the effect of reducing land use efficiency in each province, where the coefficient increased from north to south but with weak effects overall. In Heilongjiang province, the absolute value of the PS coefficient was the highest, meaning it had the strongest negative effect. The lowest value and the weakest effect were found in Hainan province. The main reasons for this were as follows: Heilongjiang, Jilin, Liaoning, and other provinces making up of the old industrial base in the northeast have relied on rapid industrialization to promote the economic development since the Reform and Opening-up. Under long-term extensive development, these provinces experienced problems such as low industry concentration and incoherent industrial distribution, meaning that the intensive utilization of land resources was low-which led to the strongest negative effect on land use efficiency. The regular improvement of transformation and upgrading measures to promote advanced development of the industry in the eastern coastal areas, including Beijing, Tianjin, the Yangtze River Delta and the Pearl River Delta, led to the reduction of this suppression. With the continuous optimization and upgrading of industrial structure in China, the coefficient may continue to change until it plays a positive role.

OT was an important indicator of the degree of opening to the outside world, and its coefficient was positive, meaning that a higher level of opening promoted the land use efficiency. The coefficient showed a decreasing trend from the southeast coast to the northwest inland areas, but the coefficient was only 0.029 and the difference was not significant. On the one hand, the degree of opening improves land use efficiency through the promotion of economic development; on the other hand, it will further promote the continuous transformation of economic development from extensive to intensive, which will in turn improve the land use efficiency. Due to the different economic development levels and land use patterns, the coefficient had a decreasing trend from the northeast to the southwest.

DMSP/OLS, DEM and NDVI are the main feature indicators of regional natural conditions. The coefficients for DMSP/OLS and NDVI were positive, and they were positive and negative for DEM. The average value of the NDVI coefficient was only exceeded by PGDP, meaning it was a significant predictor of higher land use efficiency. The coefficient for DMSP/OLS was small, implying that it had a weak positive effect on land use efficiency. The DEM coefficients led to lower land use efficiency in the contiguous region from Heilongjiang to Inner Mongolia, Shanxi, Hebei, Shandong, Jiangsu, and Shanghai, but in other areas DEM played a role in promoting the land use efficiency. The spatial distribution trends of the coefficients for DMSP/OLS, DEM, and NDVI were essentially the same, decreasing from the southwest to the northeast. The reasons were as follows: although DMSP/OLS was a representation of regional social attributes, its continuous distribution characteristics made it’s a complex of social and natural factors. DMSP/OLS, DEM and NDVI had the same distribution trend, which was closely related to the distribution of natural geographical features in China. Due to the fragile natural conditions in the west and southwest, with the increasing awareness of environmental protection, the improvement of natural resources plays a great role in the promotion of land use efficiency. The ecosystem stability in the eastern region is stronger and the effect of land use is weaker in there.

## Discussion

This study selected CO_2_ emission as an undesirable output and used the Super SBM DEA and ESDA-GWR to study the spatio-temporal variation and influencing factors on land use efficiency in 30 provinces in China from 2003 to 2013. The following conclusions can be drawn.

### Spatio-temporal variation of land use efficiency

Land use efficiency in different regions of China shows different characteristics^20,22,23^. Overall, the land use efficiency in China can be categorized as moderately ineffective, and the utilization level is low. Land use efficiency shows a positive spatial autocorrelation; global autocorrelation from 2003 to 2013 displayed an increasing trend and maintained high levels, which was consistent with the unbalanced economic development that’s high in the east and low in the midwest.

There is a clear aggregation of “hot spots” with high values and “cold spots” with low of land use efficiency. The “hot spots” are mainly distributed in the southeast coastal areas, and the “cold spots” are mainly found in the central and western regions. There is a “S” type distribution curve of “high - low” and “low - high” types in the regions of Liaoning, Hebei, Shandong, Anhui, Jiangxi, Guangxi, Yunnan, and Guangdong. Over the course of the study period, the “hot spots” and “cold spots” have gradually stabilized with an overall pattern of higher land use efficiency in the east and low in the west.

### Influence factors of land use efficiency

Land use efficiency was the result of the social and economic development factors^24–26^. Based on the GWR, the degree of each variable can be ranked as PGDP > NDVI > OT > PF > DMSP/OLS > PS > DEM. PGDP, PF, OT, DMSP/OLS and NDVI had a positive effect, while DEM had both positive and negative effects, and PS had negative effects in each province. At the same time, it was found that the continuously distributed natural factors such as NDVI, DMSP/OLS, and DEM had a significant impact on land use efficiency, and this finding would be profound for the related researches on land use efficiency.

### Implications for improving land use efficiency

The natural and socio-economic factors such as PGDP, PF, OT, DMSP/OLS and NDVI had a positive effect, therefore, we need to improve the PGDP, PF, OT and other social and economic indicators, and protecting the ecological environment and increasing the vegetation coverage to improve the NDVI level are also needed. Meanwhile, we should put efforts to improve the overall level of social development, thus to improve the regional DMSP/OLS. The PS had a negative effect on land use efficiency, therefore, all provinces need to upgrade the industry, reduce the proportion of the second industry, and strive to increase the proportion of the tertiary industry.

The research results of the land use efficiency showed the hot spots are mainly distributed in the southeast coastal areas, and the cold spots are mainly found in the central and western regions, with that the influence factors also show obvious geographical distribution difference. For the eastern coastal areas, the emphasis should be improving their OT, PF and PGDP. For the western region of less economically developed, it should focus on improving its comprehensive economic development level to improve the DMSP/OLS, while strengthening the ecological environment to improve the level of NDVI. At the same time, should actively promote energy conservation and emission reduction and increase the overall land use efficiency level.

Due to data acquisition limitations, the number of variables selected for this study is limited. At the same time, the research on the factors of influence for land use efficiency is not deep enough. In the future, we hope to consider other factors that may reveal the spatio-temporal evolution characteristics and the driving mechanisms for land use efficiency.

## Materials and Methods

### Index Selection and Data Sources

This study selected 30 Chinese provinces (some are municipalities or autonomous regions) between 2003 and 2013 to be the research unit. Because the data for Hong Kong, Macao, Taiwan, and Tibet were missing, they were excluded from the study. This study considered the model characteristics and research objectives to determine an evaluation index system (Table 5). The input indices included inputs for capital, labor, land, and energy; among these, the capital stocks could be estimated based on Zhang Jun’s perpetual inventory method^27^. The number of employees was derived from the corresponding year’s “China Statistical Yearbook,” while urban land area was derived from the “Statistical Yearbook of China’s Urban Construction”, the energy consumption data was primarily sourced from the “China Energy Statistical Yearbook”, and the total crop sown area was from the “China Rural Statistical Yearbook.” Indicators for expected outputs were selected from the gross domestic product (GDP) and adjusted in accordance with the corresponding year GDP deflation using 2003 as the base period; these data all came from the “China Statistical Yearbook”. The carbon emissions were selected to be the undesirable output index, and the data was obtained using Liu and Yan’s method^28^.

**Table 5:** Index System for Land Use Efficiency in China.

Index Type	First Grade Index	Second Grade Index
Input Index	Capital Input	Capital Stock
	Labor Input	Number of People Employed
	Land Input	Total Area of Crops Sown, Urban Construction Land Area
	Energy Input	Energy Consumption
Output Index	Economic	GDP
	Pollution	CO_2_ emissions

### Super-SBM DEA model

DEA is a non-parametric statistical method that uses a linear programming model to evaluate the efficiency of multi-input and multi-output decision-making units (DMU) of the same type^29^, and it has been widely used in transport, public infrastructure, agriculture, environmental, etc^18,30–39^. The relative effective unit efficiency of a traditional DEA model is 1, which does not resolve the sorting problem of the relative effective unit; however, the Super-SBM DEA model proposed by Tone provides a good solution^40^.

For the Super-SBM DEA model with *m* types of inputs and *s* types of outputs, the formula is as follows:

1 $$\begin{array}{rcl}{\rho }^{\ast } & = & \min \,\frac{1-\frac{1}{m}\sum _{k=1}^{m}{s}_{k}^{-}/{x}_{i0}}{1+\frac{1}{s}\sum _{r=1}^{s}{s}_{r}^{+}/{y}_{r0}}\\ {x}_{0} & = & X\lambda +{s}^{-}\\ {y}_{0} & = & Y\lambda -{s}^{-}\\ \lambda  & \ge  & 0,{s}^{-}\ge 0,{s}^{+}\ge 0\end{array}$$

In Eq. 1, ${\rho }^{\ast }$ represents the efficiency value of DMU (${x}_{0}$,${y}_{0}$), $X$ is the input columns, $Y$ is the output columns, ${s}_{k}^{-}$ represents $k$ different types of input redundancy, ${s}_{r}^{+}$ represents $r$ types of insufficient output. The value of $X$ cannot be equal to ${x}_{0}$, while the value of $Y$ value cannot be equal to ${y}_{0}$. This means that the efficiency value can be greater than 1, an effective solution to the relative unit efficiency problem described above.

### Exploratory spatial data analysis

Spatial autocorrelation analysis is an important part of the ESDA method, which includes a global spatial autocorrelation index for measuring full spatial distribution characteristics, as well as a local spatial autocorrelation index for measuring local spatial distribution characteristics. ESDA method has been widely used in different studies, such as land use^41,42^, landscape^43,44^, geochemistry^45^, environment^46,47^, medical^48^ and so on.

The Moran’s *I* index is used to measure global spatial autocorrelation, and the formula is as follows:

2 $$I=\frac{n\sum _{i}^{n}\sum _{j}^{n}{w}_{ij}({y}_{i}-\overline{y})({y}_{j}-\overline{y})}{(\sum _{i}^{n}\sum _{j}^{n}{w}_{ij})\sum _{i}^{n}{({y}_{i}-\overline{y})}^{2}}$$

where *n* is the total number of units in the study area, *y*_*i*_ *and y*_*j*_ are the attribute values of point *i*, *j*, $\overline{y}$ is the average value of all attribute values in the study area, and ${w}_{ij}$ is the spatial weight. Moran’s *I* range from [−1, 1], indicating that there is a negative correlation when the observed value is less than 0, an independent random distribution when it is equal to 0, and a positive correlation when it is greater than 0.

The local Moran’s *I* is used to represent local spatial autocorrelation, proposed in 1995 by Anselin^49^, and the formula is as follows:

3 $${I}_{1}={z}_{i}\sum _{i}{w}_{ij}{z}_{j}$$

where ${z}_{i}$ is the standard amount of the mean value, ${z}_{j}$ is the standardized quantity of the standard deviation, ${z}_{i}=\frac{{x}_{i}-\overline{x}}{\delta }$, and $\delta$ is the standard deviation of ${x}_{i}$.

### Geographically weighted regression

The GWR proposed by Brunsdon^50^, is an improved spatial linear regression model whose main advantage is that the spatial weight matrix is applied to a linear regression model. The model is capable of displaying sharp differences in spatial structure, a tool that’s widely applied in economics, geography, environmental science, and criminology^51–62^. The formula is as follows:

4 $${y}_{i}={\beta }_{0}({\mu }_{i},{v}_{i})+\sum _{k=1}^{p}{\beta }_{k}({\mu }_{i},{v}_{i}){x}_{ik}+{\varepsilon }_{i}$$

where ${y}_{i}$ represents the observed value, $({\mu }_{i},{v}_{i})$ are the coordinates of the point $i$, ${\beta }_{0}({\mu }_{i},{v}_{i})$ is the $k$ regression constant of the point $i$ and a function of geographic location; $p$ is the number of independent variables; ${x}_{ik}$ is the value of the independent variable ${x}_{k}$ at the point $i$; and ${\varepsilon }_{i}$ is a random error coefficient.

### Construction of the GWR model

The regional differences in land use efficiency were the result of the comprehensive function of many factors. Many studies researched the social and economic development factors, such as economic development, industrial structure, levels of urbanization, and levels of management and organization, but these rarely considered the natural factors. Therefore, this study was based on the full use of existing information, used DEM, DMSP/OLS and NDVI data and researched the impact of other natural factors on the land use efficiency. PGDP, PS, PF, OT, UR, DEM, DMSP / OLS and NDVI were initially considered to be the influencing factors (Table 6). In order to eliminate co-linearity, the indices were standardized and SPSS 20 was used to perform the multiple co-linear diagnosis, which ultimately led to the removal of the UR index due to the large variance inflation factor (VIF). Finally, PGDP, PS, PF, OT, DEM, DMSP/OLS and NDVI were selected to be the influence factors of land use efficiency, and the land use score of 2013 was selected as the dependent variable, so the GWR model was constructed based on formula (4).

**Table 6:** The influence factors of land use efficiency. Y means it’s selected to be the factor of land use efficiency, N means it’s removed due to its larger variance inflation factor (VIF).

Index	Description	Remark
DEM	Digital Elevation Model	Y
DMSP/OLS	Defense Meteorological Satellite Program/Operational Linescan System	Y
NDVI	Normalized Difference Vegetation Index	Y
PGDP	per capita GDP	Y
PS	the second industrial added value accounting for the proportion of GDP	Y
PF	investment in fixed assets accounting for the proportion of GDP	Y
OT	exports accounting for the proportion of GDP	Y
UR	urbanization rate	N
